# Recognizing Skin Popping Scars: A Complication of Illicit Drug Use

**DOI:** 10.7759/cureus.2726

**Published:** 2018-06-01

**Authors:** Rachael C Saporito, Mildred A Lopez Pineiro, Micheal R Migden, Sirunya Silapunt

**Affiliations:** 1 Internal Medicine, Baylor College of Medicine; 2 Dermatology, University of Texas Mcgovern Medical School at Houston, Houston, USA; 3 Departments of Dermatology and Head and Neck Surgery, The University of Texas Md Anderson Cancer Center, Houston, Texas

**Keywords:** skin popping scars, complications, illicit drug use

## Abstract

“Skin popping” is a method of injecting illicit drugs into the skin. There are numerous acute and chronic complications associated with skin popping. We present a case of a 48-year-old, African-American female patient with 40 - 60 hyperpigmented, fibrotic, depressed, round papules and plaques on the extremities, which were incidentally noticed during a clinic visit for her acne vulgaris. Skin popping scars are important clues for possible drug abuse. Healthcare practitioners should be aware of and recognize the lesions associated with this practice so further testing can be performed if clinically indicated. Recognition of the lesions and thus earlier treatment of the complications could prevent the complications of skin popping in the skin and other organs.

## Introduction

“Skin popping” is a method of injecting illicit drugs, especially cocaine, opiates, and barbiturates, into the skin with the goal of achieving slower absorption, decreased risk of overdose, and easier administration than with intravenous drug use [1]. We present a case of skin popping scars, a chronic complication of illicit drug use.

## Case presentation

**Figure 1:**
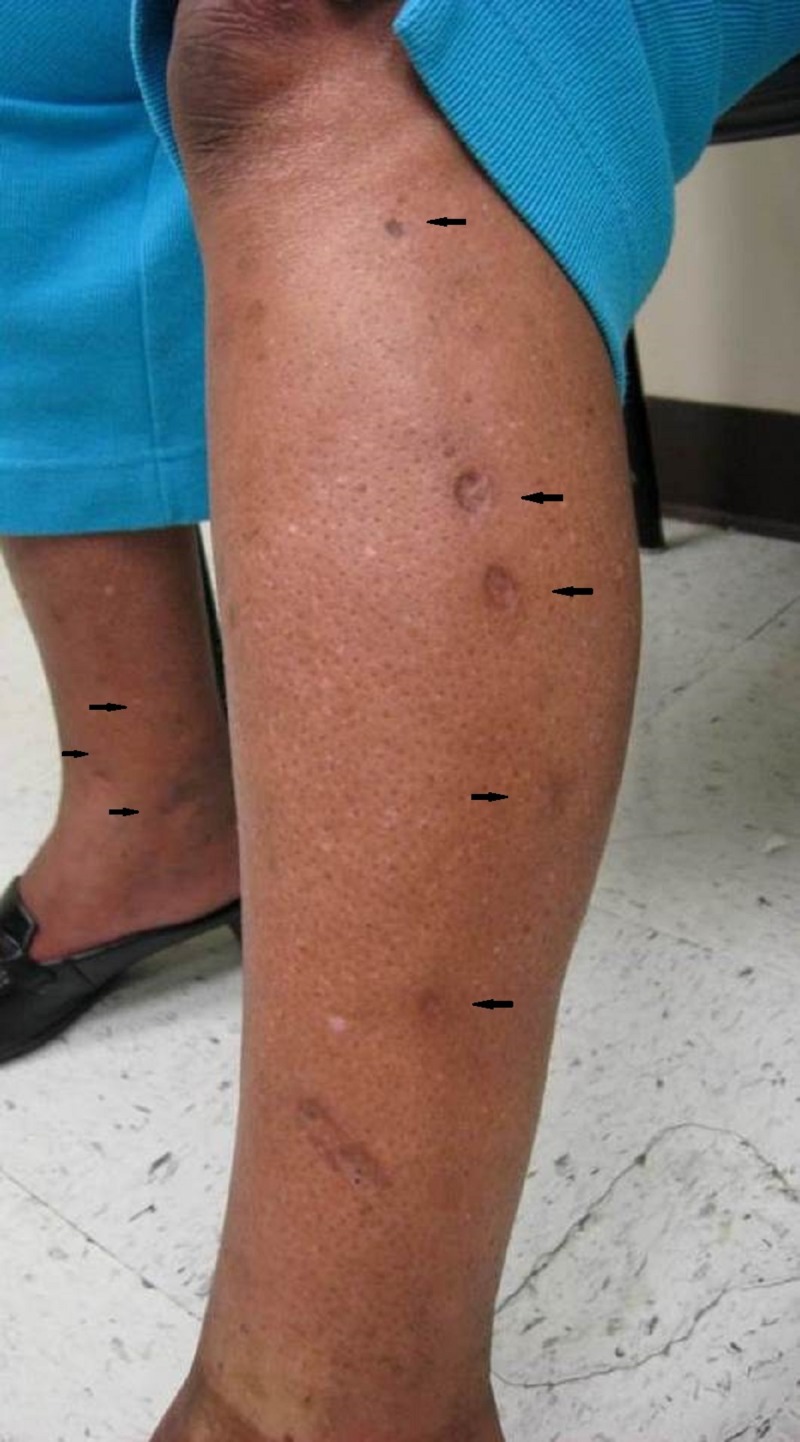
Left lower leg with fibrotic, depressed, round papules (black arrows)

**Figure 2:**
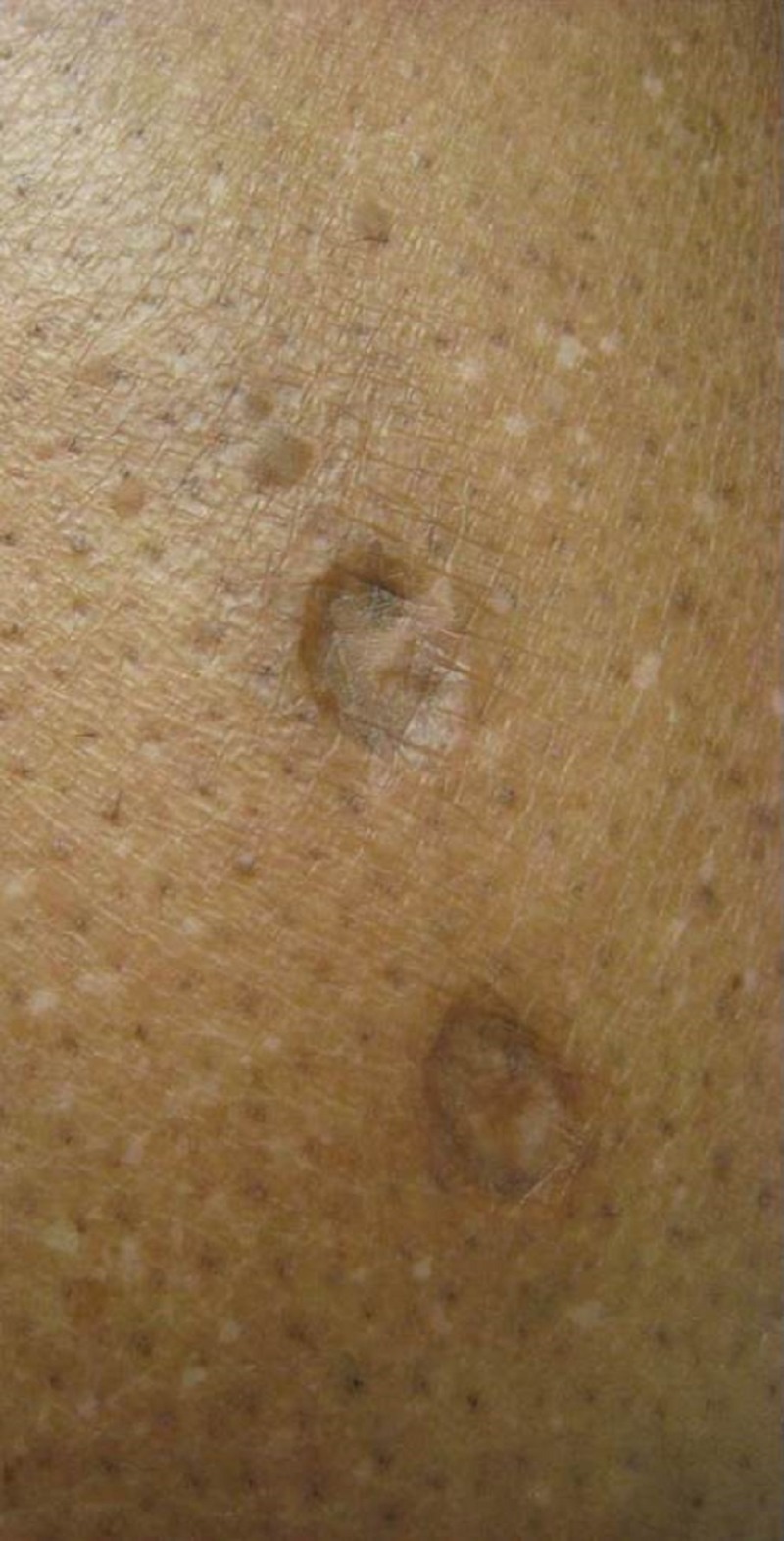
A close-up view of the skin popping scars with atrophic, hyperpigmented papules

**Figure 3:**
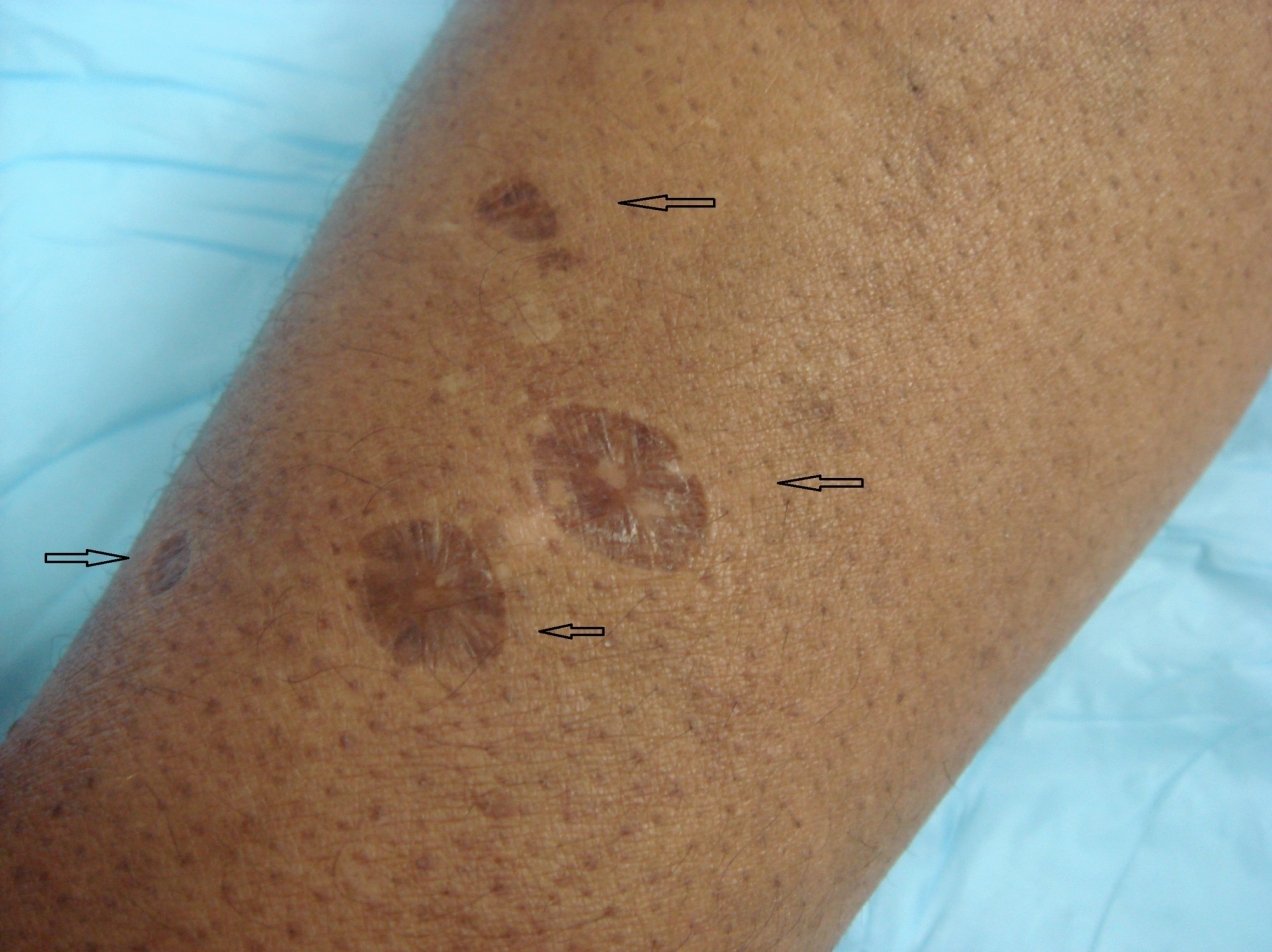
Forearm with round, depressed, hyperpigmented papules and plaques (arrows)

A 48-year-old, African-American female patient presented for treatment of acne vulgaris and was incidentally found to have 40 - 60 hyperpigmented, fibrotic, depressed, round, 5 to 15 mm papules and plaques on the forearms and lower legs (Figures 1-3). The patient revealed that these lesions were sites where she had injected heroin and that she had a 10-year history of heroin and other illegal drug use. She reported a history of recurrent abscesses and cellulitis on her extremities. Based on her clinical history and characteristic skin findings, the lesions were diagnosed as skin popping scars. She was counseled regarding her condition. She reported being drug-free for the past 20 years. No biopsy was performed, and blood tests for hepatitis B virus, hepatitis C virus, and human immunodeficiency virus were negative.

## Discussion

The most common acute cutaneous complications of skin popping are bacterial infections, including abscesses and cellulitis [2-3]. Skin popping allows direct inoculation of bacteria and irritants into the skin, and as a result, use of this method has the greatest risk factor for the formation of suppurative skin infections versus other routes of administration [2-3]. A study showed the odds of abscess or cellulitis among drug users using skin popping was almost five times the odds of those who used intravenous injection [4]. The most common bacteria cultured from these skin infections is Staphylococcus aureus either alone or in combination with anaerobic bacteria [2-5].

Other infections can occur, such as Candida folliculitis, botulism, tetanus, and necrotizing fasciitis [2, 4-7]. Self-treatment among this population with lancing of abscesses and antibiotics purchased on the street is common, which presents further complications due to lack of sterilization and potentially improper use of antibiotics [4]. 

Chronic complications include scar formation (as seen in our patient), hyperpigmentation, cutaneous granulomas from contaminants (such as talc), and even necrosis of the digits if vasoconstrictive substances (such as cocaine) are incidentally injected into small arteries (Lopez-Pineiro M, et al.: Skin popping scars: A chronic complication of illicit drug use (abstract-AB237) Presented at the 75th Annual Meeting of the American Academy of Dermatology, Orlando, FL, March 3-7, 2017. doi: 10.1016/j.jaad.2017.04.918) [2, 8]. Moreover, cases have been reported of serum amyloid A amyloidosis in patients with histories of skin popping, which can lead to renal impairment (Table 1) [9-10]. This is thought to be due to the chronic inflammation associated with infection from skin popping. Thus, a differential diagnosis for renal insufficiency in a patient with findings of skin popping scars should include secondary amyloidosis, although consideration of this diagnosis is often overlooked [10].

**Table 1:** Complications of Skin Popping

Acute	Chronic
Infection	Scar formation
Cellulitis	Hyperpigmentation
Abscess	Cutaneous granuloma
Folliculitis	Necrosis of the digit
Necrotizing fasciitis	Serum amyloid A amyloidosis
Skin irritation	Renal insufficiency

## Conclusions

Our case highlights the importance of recognizing the lesions associated with skin popping. Patients may not be aware of the potential acute or chronic complications of such a practice. Further investigation and screening for bloodborne pathogens may be necessary. With education of patients, physicians could prevent both acute and chronic complications of skin popping on the skin and other organs.

## References

[1] Grunebaum A, Skupski D (2012). Skin popping scars - a telltale sign of past and present subcutaneous drug abuse. Case Rep Perinat Med.

[2] Gontijo B, Bittencourt FV, Lourenco LFS (2006). Skin manifestations of illicit drug use (article in Portuguese and English). An Bras Dermatol.

[3] Ebright JR, Pieper B (2002). Skin and soft tissue infections in injection drug users. Infect Dis Clin North Am.

[4] Binswanger IA, Kral AH, Bluthenthal RN (2000). High prevalence of abscesses and cellulitis among community-recruited injection drug users in San Francisco. Clin Infect Dis.

[5] Summanen PH, Talan DA, Strong C (1995). Bacteriology of skin and soft-tissue infections: comparison of infections in intravenous drug users and individuals with no history of intravenous drug use. Clin Infect Dis.

[6] Leclerc G, Weber M, Contet-Audonneau N, Beurey J (1986). Candida folliculitis in heroin addicts. Int J Dermatol.

[7] Vugia DJ, Werner SB, Woodfill CJ (2004). Wound Botulism, Tetanus, and Necrotizing Fasciitis among Injecting Drug Users in California: the Clostridial Connection. Emerging Infections 6.

[8] Del Giudice P, Vandenbos F, Boissy C (2006). Cutaneous complications of direct intra-arterial injection in drug addicts. Acta Derm Venereol.

[9] Cooper C, Bilbao JE, Said S (2013). Serum amyloid A renal amyloidosis in a chronic subcutaneous (“skin popping”) heroin user. J Nephropathol.

[10] Nayer A (2014). Amyloid A amyloidosis: frequently neglected renal disease in injecting drug users. J Nephropathol.

